# No detectable effect of urbanization on genetic drift or gene flow in specialist herbivorous insects of milkweed

**DOI:** 10.1371/journal.pone.0318956

**Published:** 2025-02-14

**Authors:** Lindsay S. Miles, Elizabeth J. Carlen, Zain Nassrullah, Jason Munshi-South, Marc T. J. Johnson

**Affiliations:** 1 Department of Biology, University of Toronto Mississauga, Mississauga, Ontario, Canada; 2 Department of Entomology, Virginia Polytechnic Institute and State University, Blacksburg, Virginia, United States of America; 3 Living Earth Collaborative, Washington University, St. Louis, Missouri, United States of America; 4 Department of Biological Sciences, Fordham University, New York City, New York, United States of America; University of Saskatchewan College of Agriculture and Bioresources, CANADA

## Abstract

Urbanization is hypothesized to isolate populations and restrict dispersal, leading to reduced genetic diversity and increased genetic differentiation. We tested this hypothesis in specialist herbivorous insects of milkweed, positing that higher dispersal ability would mitigate the negative effects of urbanization on genetic drift and gene flow, and that these effects would vary with city size. In this study, we collected 383 milkweed insects from urban and rural sites in Toronto, Canada, and five surrounding cities. Using ddRADseq, we generated 145,000 SPNs for monarchs, 10,000 SNPs for beetles, 6,000 SNPs for weevils to quantify genetic diversity, demographic history and population genetic structure. Contrary to our hypotheses, our results indicated no effect of urbanization or dispersal ability on diversity or genetic differentiation. Genetic diversity, measured as π, varied between 0.0013 and 0.0044 across species, with no urban vs. rural component, but with monarchs having >2 X higher diversity compared to beetles and weevils. Similarly, genetic differentiation was generally low, F_ST_ varying between 0.01 and 0.28, but there are no consistent trends among urban vs. rural samples for any of the three species. However, demographic analyses revealed a consistent decline in effective population size for all three sampled species, beginning around the last glacial maximum and intensifying over the past 1,000 years. Our findings suggest that both urbanization and dispersal ability have not been a major factor in reducing gene flow or increasing genetic drift among milkweed’s herbivorous insect populations. Instead, historical events such as climatic change since the last glacial maximum, and large-scale anthropogenic disturbance in general, have had a more pronounced impact on demography. These results highlight the importance of considering the combined effects of natural and anthropogenic long-term historical processes when studying population genetics in the context of urbanization.

## Introduction

Urbanization is a global phenomenon that is rapidly transforming rural landscapes into buildings, roads and related infrastructure to support high densities of people [1,2]. The development of cities results in increased habitat fragmentation and degradation [3] that can influence evolutionary processes within and around urban landscapes [4–6]. Fragmented urban habitats can isolate populations within habitat patches, leading to increased genetic drift within surviving populations and reduced gene flow between populations [7,8]. These populations are often at higher risk of local extinction because of lower genetic diversity within populations, increased inbreeding, and decreased capacity for adaptive evolution.

It is increasingly recognized that urbanization can have large impacts on nonadaptive evolutionary dynamics (i.e., genetic drift and gene flow), but it remains unclear whether these effects occur in parallel across cities and among species [9]. The human-built environment shows many commonalities across cities, including high human population densities, increased impervious surfaces (e.g., roads and buildings), higher habitat fragmentation, elevated pollution (e.g., air quality and light), and elevated temperatures (i.e., urban heat island) [10,11]. Therefore, cities are expected to drive parallel evolution as urban populations experience these similar environments [12]. However, cities are not always homogeneous and can vary in size, age, design, urban infrastructure, and local climate [3,13]. In fact, cities can exhibit substantial heterogeneity within their landscape [13–15], which can change genetic variation across a cline [12]. Such heterogeneity among cities may alter long-term population demography and dispersal, with consequences for genetic drift and gene flow. Species may show stronger reductions in genetic diversity within populations and greater divergence between populations across larger, more intensely urbanized cities, whereas smaller cities may allow gene flow between urban and rural populations that maintains genetic diversity and reduces differentiation [16]. These patterns of genetic drift and gene flow may be dampened when comparing larger and smaller cities that are in different geographic regions, as environmental conditions might have stronger influences on evolution than anthropogenic effects. Therefore, comparing cities of different sizes within the same geographic regions may facilitate understanding the effects of urbanization on landscapes genetic variation.

To understand how urbanization affects the influence of genetic drift and gene flow on population divergence, we must consider dispersal strategies and their consequences. Restricted dispersal is one of the primary explanations for increased genetic divergence between populations [17–19]. This restricted dispersal can be due to landscape barriers (e.g., urban habitat fragmentation) or relative mobility (e.g., walking vs flying). Generally, dispersal type predicts genetic divergence in vertebrates, where flying animals tend to have lower genetic differentiation than walking animals [20]; but see [8]). However, dispersal type and distance has not been a predictor of invertebrate genetic differentiation [21], likely due to the additional influences of life history variation [22]. Invertebrates that occupy the same ecological community, but with varying dispersal abilities may provide novel insight into the interplay between urbanization and dispersal ability in shaping patterns of genetic differentiation. The dispersal ability of organisms that live near cities has a strong influence on whether they will colonize urban green spaces, where insects with low dispersal ability are expected to be more negatively affected by the urban matrix, thus reducing gene flow [7,23,24]. Insects with low dispersal ability are expected to be sensitive to urban fragmentation and thus have higher genetic differentiation, whereas those with high dispersal ability will be more likely to overcome urban disturbance and have generally lower genetic differentiation.

We must also consider the historic demography of each species because it shapes contemporary effective population size and thus standing genetic variation. Specifically, we must examine how populations have been shaped by prior global climate events. The landscape and climate of North America, especially Canada, was greatly influenced by the last glacial maximum approximately 20,000 years ago. The extension of glaciers across Canada led many taxa to retreat from the cooler poles of the earth towards refugia at lower latitudes [25,26]. The subsequent retreat of glaciers as Earth began to rewarm led to species recolonizing higher latitudes. These large-scale events are particularly important to consider when studying present-day population dynamics as they shape the pool of diversity that modern evolutionary forces may act upon.

Specialized insect herbivores provide an excellent model to examine how urbanization affects genetic drift and gene flow. Specialized insect herbivores are expected to be particularly sensitive to urbanization because of their restricted diets. Thus, any effect of urbanization on the size and connectivity of host populations is expected to have cascading effects for insect species that rely on them. The specialist insect community of the common milkweed (*Asclepias syriaca*) has long served as a model for the ecology and evolution of plant-herbivore interactions [27–31]. The community ecology and phenology of these insects have been thoroughly studied in rural habitats [32–36], but their evolution in urban habitats is still understudied. Previously we investigated how urbanization affected the diversity, composition and abundance of insect herbivores across urban-rural habitats across multiple cities that varied in size. We found that total abundance and species richness often differed between urban and rural habitats [37]. This difference in abundance was due to the presence of specific species like the leaf mining fly that was most abundant in urban habitats, whereas the milkweed weevil was more abundant in rural habitats. Monarch butterflies were found at near equal abundance in urban and rural habitats, which suggests that dispersal ability may mitigate negative consequences of urban barriers. Based on these ecological findings, we expect genetic diversity and differentiation to be most affected in the low dispersing milkweed weevil and least affected in high dispersing monarch butterflies.

Here, we test the hypothesis that urbanization influences genetic drift and gene flow. However, since gene flow depends on the movement of individuals across a landscape, we expect that the effects of urbanization will depend on dispersal ability of the focal species. Likewise, since genetic drift is a function of population size, the effects of urbanization on this neutral evolutionary process should depend on the size of a city, since larger cities are expected to repress and isolate populations to a larger degree than smaller cities. Specifically, we predict: 1) decreased dispersal ability of milkweed’s specialist insect herbivores will amplify the negative effects of urbanization on gene flow and genetic drift. 2) Effects of urbanization on genetic drift and gene flow will be qualitatively consistent among cities, but the magnitude of the effect will increase with city size. We test these hypotheses across three milkweed herbivore specialists in six cities in southern Ontario, Canada.

## Methods

### Study system

**Fig 1:**
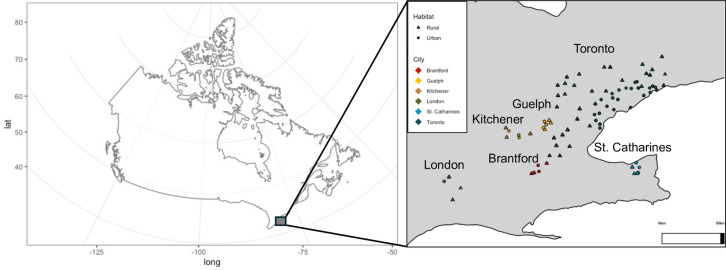
Sampling map of all species across 6 cities. Monarchs, beetles, and weevils were collected from urban and rural sites that contained milkweed plants. Each color represents a sampled city on the map, rural sites (outside of city boundaries) are triangles and urban sites (within city boundaries) are circles.

Common milkweed is distributed across eastern North America in natural (e.g., prairies, wetlands) and disturbed (e.g., lawns, roadsides) grasslands [38,39]. All *Asclepias* spp. produce antiherbivore defenses, which include cardenolides and latex [40–43] that limit herbivore species to a small community of specialist insects that have adapted to these plant defenses [28,30,44–46]. In our study region, the common milkweed, A. syriaca, is most prevalent. While there are 10 specialist insect herbivores that commonly occur specifically on *A. syriaca* in our area (see field sampling below Fig 1), here we focus on three insects that have varying dispersal abilities—monarch butterflies (*Danaus plexippus*, Lepidoptera: Nymphalidae), longhorn milkweed beetles (*Tetraopes tetrophthalmus*, Coleoptera: Cerambycidae) and milkweed weevils (*Rhyssomatus lineaticollis*, Coleoptera: Curculionoidea) – for brevity hereafter referred to as monarch, beetle, and weevil, respectively. Monarch adults in our study region migrate through flight >1000 km [47,48] and are found in abundance in urban and non-urban habitats [37]. While there is a small non-migratory monarch population in Southern California, this location is far from our study region and we can therefore assume that our monarch population consists solely of migratory individuals [49]. Beetles are also able to disperse through flight and travel up to 30–40 km with short bursts of 1–2 km [50,51]. Weevils are mostly flightless and are only able to disperse up to 15 m [50] and are generally found in higher abundance in rural habitats compared to urban habitats [37].

### Sampling

During the summer of 2019 we collected monarch caterpillars, beetle adults, and weevil larvae and adults from A. syriaca plants in urban and rural habitats across six cities. We hand captured each individual and placed it in a falcon tube before transporting it to the laboratory and freezing at -20°C. All samples were collected on private property with permission from owners, from municipal roadsides, and from municipal parks, thus no collection permits were required. All sampling locations are located in southern Ontario and vary in both geographic and human population size by over two orders of magnitude (S1 Table). All cities studied were established within 84 years of one another, from 1793 (Toronto) to 1877 (Brantford). Each sampling locale was a minimum of 500 m away from the nearest site to avoid sampling related individuals. To identify the fine scale effects of urbanization within and between cities, we sampled 130 locales, with 2–10 individuals collected per locale. In the Greater Toronto Area (hereafter “Toronto”), we sampled 50 urban and 50 rural locales (Fig 1). Toronto is one of North America’s largest metropolitan areas, spanning the largest geographic extent of the six cities sampled here. To identify the effects of urbanization among cities, we collected individuals from each species from an additional 5 cities in Ontario, Canada – Brantford, London, Guelph, St. Catharines, and Kitchener – which included three sampling locations inside the city boundary (“urban”) and three sampling locations outside the city boundary (“rural”) (Fig 1). The sampling map was generated in R v.4.3.0 using maptools v1.1-8 [52] and ggplot2 v3.4.4 [53].

### DNA extraction, ddRAD-seq library preparation, and sequencing

We dissected approximately 20 mg from the head region for both beetle and monarch samples to avoid gut microbes and fatty tissues and used whole body tissue for weevils*,* as these individuals were <25 mg. We extracted DNA using the Qiagen DNeasy Blood and Tissue Kits (Qiagen, Inc.) following the manufacturer’s protocol with one additional step; we added 4 µl of RNase A after digestion, to remove potential downstream RNA contamination. We quantified the DNA concentration using a Qubit fluorometer with the dsDNA Broad Range Assay Kit (Thermo Fisher Scientific), then used individuals with at least 1000 ng DNA for ddRADseq library preparation.

We prepared ddRAD libraries following the protocol outlined in Peterson et al. [54]. Briefly, we digested DNA with restriction enzymes, ligated barcode adapters, size selected pooled samples, then PCR amplified pools, with bead cleaning between steps. We selected SphI and MluCI enzymes because they provided ample sequences within our target range of 376–412 bp for each of the three species according to the ddRADseq_inSilico python script (https://github.com/James-S-Santangelo/ddRADseq_inSilico). In total, we digested 425 samples that were combined into nine pools of 48 and one pool of 41. These pools were then sequenced on a single lane of a NovaSeq 6000 at Novogene using paired-end 125 bp sequencing.

### SNP calling, data filtering

Sequence reads were demultiplexed using the *process_radtags* program in Stacks v2.64 [55] using the parameters −q and −r (discard reads with low quality scores and rescue barcodes, respectively).

The monarch reads were aligned to the *Danaus plexippus* genome (NCBI accession GCF_018135715.1) using default parameters in Bowtie2 2.4.2 [56,57]. The SAM files were converted to BAM files using SAMtools [58], then reads were further processed with the *gstacks* program in Stacks using default parameters. Because monarch butterflies can lay multiple eggs on a single plant and across multiple sites [59,60], and only a few individuals were collected per locale, highly related individuals within and between sites are possible. Highly related individuals can influence population genetic structure analyses [61–63], therefore, we used the program *VCFtools* v0.1.16 [64] with the—relatedness flag to identify related individuals of monarchs. The expectation is that the Ajk statistic [65] will be zero for unrelated individuals and 1 for an individual with itself. We randomly removed one individual per locale when their relatedness was greater than 0.75 and removed an additional individual that was related (Ajk = 0.5) to multiple individuals from 5 locales.

Since, there are no reference genomes or closely related species’ genomes available for the beetle or weevil, we used the *de novo* pipeline of Stacks. Additionally, we assumed that both species were diploid as supported by Smith [66] for T. tetrathalamus, and Lachowska et al. [67] for Rhyssomatus. The *ustacks* program in Stacks was first optimized for the M (distance allowed between stacks), m (minimum stacks depth) and n (distance allowed between catalog loci) parameters. This optimization identifies the combination of parameters that maximizes the number of “real” RAD loci [68,69]. We randomly subsampled 20 individuals per species and iterated through the entire de novo pipeline with m =  3 to m =  7 (M and n were kept to default values), picking the most well supported m value. We then repeated this process with M =  1 to M =  8, picking the most well supported M, and finally repeating with n =  M – 1 to n =  M +  1. For the beetle sequence data, we found the most well supported values were m =  6, M =  2, and n =  1, which were then used in subsequent analyses. For the weevil sequence data, we found the most well supported values were m =  5, M =  2, and n =  1. For each of the two species, the catalog of RAD loci was generated with *cstacks* by randomly subsampling 20 individuals. Next, loci from all individuals were matched to the catalog using *sstacks* with default parameters, then the resulting tsv files were converted to bam files using *tsv2bam*. We used the *gstacks* program to incorporate the paired end reads with the --rm-unpaired-reads (discards unpaired reads) parameter.

For each of the three species, the results from *gstacks* were then further processed in the *populations* program in Stacks using the same parameters. Each individual was assigned to their sampling locale in the *popmap*, with loci present in at least 50% of individuals (--R 0.5), global minor allele frequency of 5% (--min-maf 0.05), and one random SNP per stack.

### Data analyses

#### Genetic diversity and demography.

We used the *populations* program in Stacks to calculate nucleotide diversity (π) for each species. To compare urban vs rural populations, in R we estimated the mean and standard deviation of each sample locale’s π estimate based on habitat type, compared π between species, habitat type, and city with a linear regression and a test for significance on the regression with an ANOVA, then visualized the plots using the R package *ggplot2*.

To identify patterns of demography that may have been influenced by urbanization, we conducted a stairway plot analysis [70] that models changes in effective population size over time. The unfolded site frequency spectrum (SFS) was estimated in R using the *vcf2sfs* package for each species [71], which was then used as the input file for *stairwayplots2* [70]. In the *stairwayplot* blueprint file, we used default parameters unless specified with the following exceptions: first, we set the mutation rate to 2.8E-9, which is commonly used for insect demographic analyses (e.g., [72,73]). Second, we set the monarch butterflies generation time to 4 per year because monarchs can have 3–5 generations per year [74]. Finally we set the generation time for the weevils and beetles to one per year because these species are non-migratory residents, and only breed in the summer. Time units were scaled by dividing the number of generations per year (e.g., 3 generations is ⅓). After running this analysis, we visualized the stairway plots in R using *ggplot2*.

#### Population genetic structure analyses.

To identify the effects of urbanization on population genetic structure, we used complementary methods for each of the three species. For each species we independently calculated pairwise F_ST_ [75] between each sampled locale using the *hierfstat* v 0.5-11 [76] package. Next, we calculated the mean and standard deviation of habitat pairs (rural-rural, urban-urban, urban-rural) for each species to then visualize these pairwise-by-locale F_ST_ values using *ggplot2* in R.

To identify the potential clustering across our sampling range, we used a clustering model in ADMIXTURE v1.3.0 [77]. ADMIXTURE uses a maximum likelihood model approach to estimate ancestry and provides a cross-validation score to indicate the most well supported K value. For each species we ran five iterations of K =  1–7 to account for each of the six cities sampled. Then, we used the R package *ggplot2* to visualize stacked bar plots for each of the K values. To evaluate if similar genetic clusters were identified using a multivariate approach, we performed a Discriminant Analysis of Principal Components (DAPC) with the R package *adegenet* [78]. We used the *find.clusters* function to calculate the Bayesian Information Criterion (BIC) to select the most well supported number of genetic clusters (K), then used the *optim.a.score* function to select the number of principal components to retain. We then visualized the DAPC using the *scatter* function. To further evaluate genetic clustering, we used a model-free approach and performed a Principal Component Analysis (PCA). Specifically, we used the *SNPRelate* v1.34.1 [79] R package to carry out a PCA of the vcf files then visualized the plots using the *ggplot2* R package.

## Results

We collected a total of 104 monarchs, 190 beetles, and 89 weevils across urban and rural sites in 6 cities. However, we were unable to collect any weevil samples from St. Catharines, or from urban sites in London and Kitchener. Additionally, we were unable to collect beetles from London, or urban sites in Kitchener.

### Sequence assembly

After DNA extraction, ddRADseq library preparation, and sequence filtering, we retained 1-3 individuals from each sampling locale, with>  10X coverage on average for each individual sequenced (Table 1).

**Table 1:** Sequencing assembly information for each species after site filtering.

Species	n	Total reads	Number of variant sites	Average site coverage
Monarch *Danaus plexippus*	104	42533451	145178	10.65
Beetle *Tetraopes tetrophthalmus*	190	6423312	10042	10.22
Weevil *Rhyssomatus lineaticollis*	89	3216521	6607	13.31

### Genetic diversity and demography

**Fig 2:**
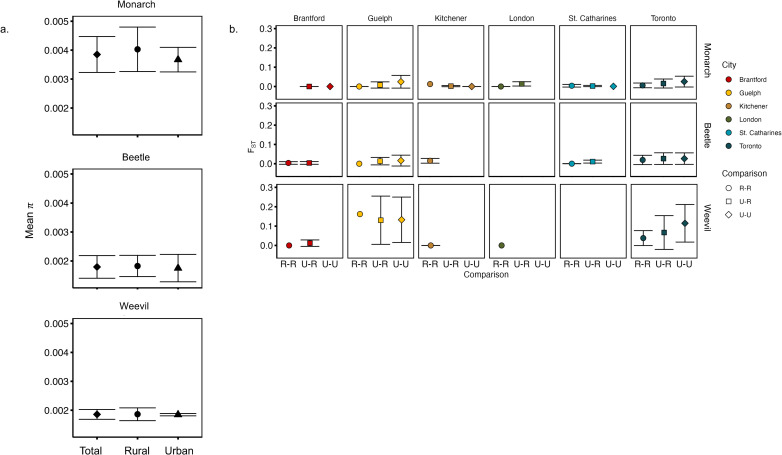
Genetic diversity among populations and genetic divergence between populations. a. In each of the three species, π was estimated for each sampling locale, separated by habitat type. There was no significant difference in π among urban and rural sites for any of the three species. b. F_ST_ was calculated between each sampling locale and there was no significant difference between any comparison type (e.g., urban vs. rural) in any city or in any species.

When comparing measures of genetic variation among species with differing dispersal abilities and between habitat types and cities, there were no clear patterns associated with urbanization. Specifically, measures of π varied between 0.0013 and 0.0044 across species. We found that “habitat” (i.e., urban vs. rural) did not influence π within each species (F_1, 23_ =  3.06e-04, p =  0.986). However, π varied among species (F_2, 23_ =  63.57, p <  0.001), with monarch having 2.15 x and 2.08 x higher mean π than either beetle or weevil, respectively (Fig 2a).

**Fig 3:**
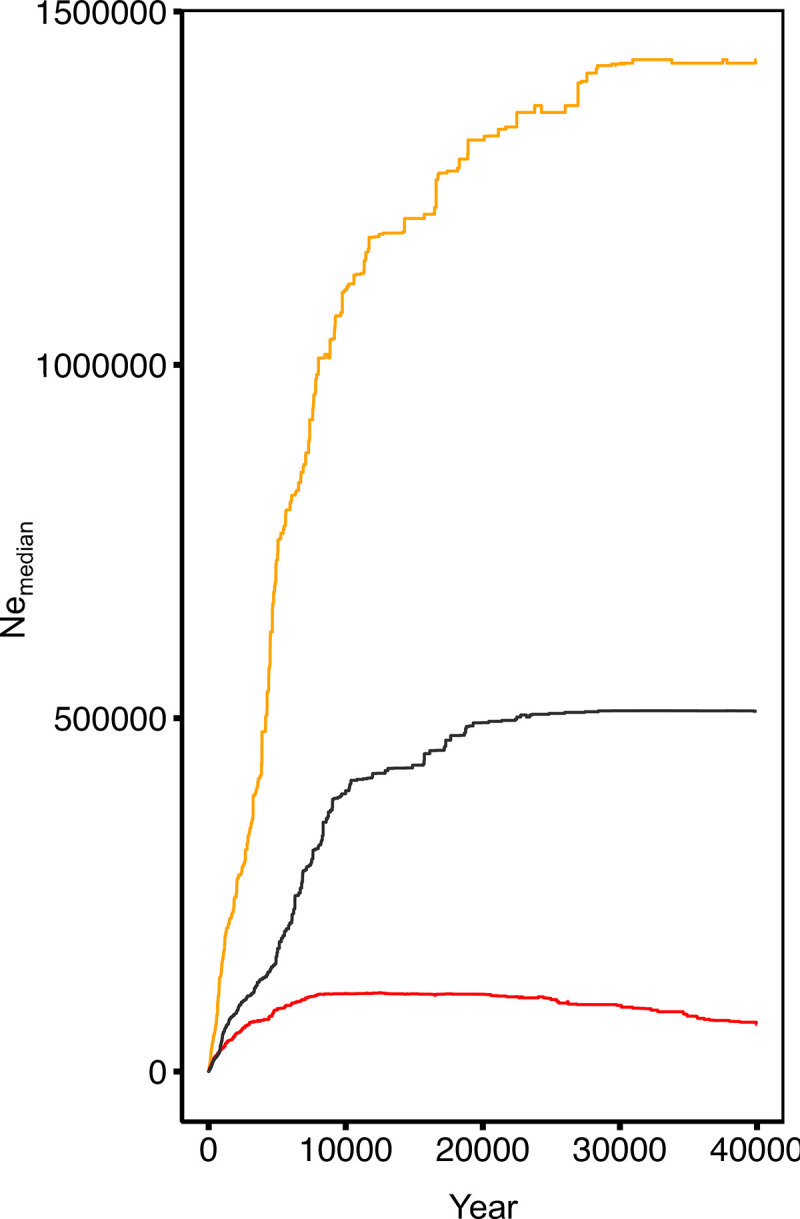
Stairway Plot. Stairway analyses of changes in effective population size (Ne) based on the site frequency spectra (SFS) for monarch (orange), beetle (red), and weevil (dark grey).

All species showed a precipitous decrease in N_e_ in the recent past, regardless of habitat. There were no differences in N_e_ between urban and rural habitats for any species (S1 Fig), so we focus our interpretation on the overall change N_e_ for each species (Fig 3). Each species shows a peak in N_e_ during the last glacial period, followed by a decline in N_e_ that started ca. 5,000 years ago in the monarch, 20,000 years ago in the beetle, and 8,000 years ago in the weevil. While this decline started slowly initially, each species exhibits a precipitous decline in Ne, especially during the past 750 years (S2 Fig).

### Population genetic structure

**Fig 4:**
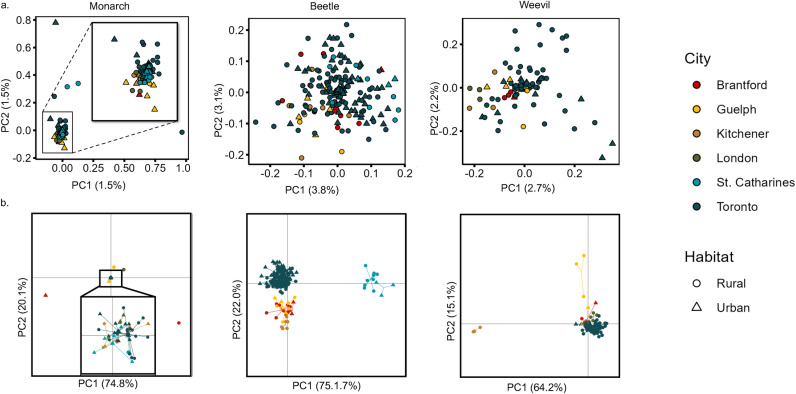
Principal Component Analysis (PCA) and Discriminant Analysis of Principal Components (DAPC). The (a.) PCA is a model free test to show individual clustering whereas the (b.) DAPC uses the population assignment to show population clustering. For the monarch, there are outliers in both the PCA and DAPC that hide the remaining clusters, so in each analysis there is also an inset of the remaining samples zoomed in. For each of the three species, there is no clustering strictly based on habitat type or city.

When comparing genetic structure between sites for the monarch, the overall F_ST_ values were low (<0.05) between any two locales. There was a slight increase in F_ST_ values when comparing urban to rural and urban to urban populations. However, this trend is only seen in two of the six cities, Guelph and Toronto, and the 95% CI are overlapping in all cases (Fig 2b). The PCA and DAPC showed no clear differentiation between urban and rural sampled individuals, regardless of the city sampled (Figs 4 and S3). The admixture analysis could not reject K =  1. When K =  2, there was no clear genetic differentiation between urban vs rural samples and when K =  6, there was no clear structure among sampled cities (S4 Fig).

When comparing genetic structure among sites for the beetle, the overall F_ST_ values were <  0.1 between any two locales. There is no discernable difference among F_ST_ values between any locale type, regardless of the city sampled (Fig 2b). The PCA does not show any separation by habitat type or by city (Figs 4a and S3), although the DAPC separates Toronto and St. Catharines from the other cities, but not by habitat type (Fig 4b). The admixture analysis supports K =  5, however individuals are assigned to multiple clusters that are unrelated to the city or habitat sampled (S4 Fig).

Finally, when comparing genetic structure among sites for the weevil, the F_ST_ values were higher than those of the beetle and monarch, with F_ST_ varying between 0.01 and 0.28. For the two cities that had multiple sampled urban and rural locales, F_ST_ is lower among urban locales than rural locales in Guelph, but higher in urban compared to rural locales in Toronto (Fig 2b). The PCA and DAPC showed no clear differentiation among urban or rural locales (Figs 4 and S3), however individuals from the city of Kitchener are highly separated from all others (Fig 4b). The Admixture analyses supported K =  1, and similar to monarchs when K =  2, there was no separation among urban vs rural samples; when K =  6 there was no clear differentiation among cities (S4 Fig).

## Discussion

Our study shows that urbanization does not have strong effects on either genetic diversity or differentiation of three specialist insect herbivores sampled across six cities. We draw our conclusion based on three results. First, all three species had equivalent levels of genetic diversity between urban and rural habitats, although species varied substantially in their level of genetic diversity. Second, species had consistent differentiation between populations regardless of habitat, although the degree of differentiation varied among cities and species. This lack of differentiation among urban and rural habitats was further supported by analyses of population genomic variation using multivariate ordinations and structure analysis. These results were qualitatively consistent across cities that varied in human population size and geographic extent by more than two orders of magnitude. Our results suggest that recent urbanization has no discernible effect on genetic drift and gene flow for any of the three species, despite large effects of urbanization on the abundance of weevils [37]. Finally, longer term demographic analyses suggest that all three herbivore species have undergone declines in N_e_ over the past several thousand years, which appear to have accelerated with historical European colonization and more recent urban development in the region. This long-term demographic result suggests that a combination of colonization of each species during post-glacial range expansion, and anthropogenic disturbance in general may have depleted genetic variation to the point where there is relatively little variation in contemporary populations. Thus, our results suggest that a combination of natural and anthropogenic processes shape the demography of specialist herbivore populations.

### Effects of urbanization on genetic drift and genetic diversity

Urbanization had no discernible effect on genetic diversity across three herbivore species or among six cities. This lack of an effect suggests that urbanization does not influence genetic drift of the species examined. This conclusion is surprising for at least two reasons. First, the herbivores of common milkweed are highly specialized, only feeding on *Asclepias* species, and in our region largely *A. syriaca* [33,80]. *Asclepias syriaca* itself is patchy in its distribution, and urban populations are particularly fragmented and isolated with only a few plants in a patch, whereas in rural habitats there are often hundreds of plants per patch [37,81,82]. Any insect on a milkweed plant must first find the plant, which we expect is particularly difficult for insects with limited dispersal ability in urban areas. Our past ecological results suggest that urbanization reduced the abundance of weevils by 41% on average between urban and rural locations, while urbanization did not influence the abundance of monarchs or beetles [37]. This ecological result corresponds to differences in dispersal ability of the three insect species, in which weevils disperse the shortest distances, beetles disperse intermediate distances, and monarchs famously disperse long distances [37]. These differences in dispersal distance and abundance with urbanization did not translate into effects on genetic diversity, N_e_ or population differentiation between urban and rural habitats. Thus, our results do not support our hypothesis that urbanization drives reduced genetic diversity and increased genetic differentiation.

Our results contrast with other studies that have found clear effects of urbanization on genetic diversity for both vertebrates and invertebrates. Miles et al. [7] found that of >150 estimates of genetic diversity in urban and rural habitats across diverse species, 94% of them showed an effect of urbanization on genetic diversity. Of the studies included, 62% found reduced genetic diversity in urban areas, consistent with the urban fragmentation hypothesis. For example, in *Peromyscus*, genetic diversity was 50% lower in the most urban sites compared to the most natural sites [83]. Still many studies (32%) found higher genetic diversity in urban areas, consistent with the urban facilitation hypothesis. For example, spring peeper frogs exhibited the highest diversity in urban populations [84]. Our results do not support either of these hypotheses, but instead support the null hypothesis of no effect of urbanization on genetic diversity.

While our results do not support the majority of studies, there are interesting parallels with other studies that have found no significant effect of urbanization on genetic diversity. Schmidt et al. [8] found that birds and amphibians [84] on average show no change in genetic diversity or N_e_ between urban and nonurban populations. Similarly, a global study of white clover (*Trifolium repens*) found no overall difference in genetic diversity of N_e_ between urban and rural populations across 24 cities, although rural populations were more likely to experience a decline in N_e_ in the recent past [12]. The pattern that emerges is that the effects of urbanization on genetic drift and its effects on genetic diversity are highly idiosyncratic, depending on the species, taxonomic group examined, and specific cities investigated. We expected that the dispersal ability and size of cities would help to resolve such variation, but our predictions were not supported by our results.

### Long-term changes in effective population size

The most striking results from our study were the parallel precipitous declines of N_e_ for each of the species during the past several thousand years. All three species exhibited high N_e_ at or preceding the last glacial maximum ca. 20k years ago, which implies that these specialist insects of milkweed maintained large populations in glacial refugia. Declines in population size start from ca. 20K years ago (monarch and weevil) to ca. 10K years ago (beetle). Notably, glaciers started their retreat from Eastern and Midwest USA 18,000 years ago and retreated from southern Ontario approximately 12,500 years ago [85]. Insect populations would have gradually tracked milkweed populations as they recolonized temperate grasslands. The time of this range expansion approximately aligns with when N_e_ is estimated to have declined for each species. Since species frequently have lower genetic diversity at their range margins [86], especially during population expansion [87], it seems likely that the reduced N_e_ for the first several thousand years after glacial retreat is explained by the effects of founder events on genetic diversity associated with a post-glacial range expansion. However, data from more southerly populations would be needed to fully address this question.

While founder events during rapid range expansion post-glaciation are likely to explain the parallel reductions of Ne, the continued precipitous declines in N_e_ during the past several hundred years are unlikely to be explained by such patterns. Instead, the recent rapid declines in N_e_ are more likely to be explained by European colonization that led to large scale land conversion to farmland starting in the mid-1800s, with increasing pesticide application in areas that remain agricultural and increasing urbanization during the past century. Early European settlers were required to clear forest cover for farming in the study area, rapidly converting southern Ontario from a mostly forested landscape to open farmland by the early 1900s [88]. While the opening of the landscape may be expected to promote the spread of the insects’ milkweed hosts, milkweed was listed as a noxious weed that was actively eradicated in Ontario until it was removed from the list in 1990 (ontariocanada.com/registry). The reduction of the host would be expected to have bottom-up effects on any insects that relied upon it. Stunningly, there is also a clear precipitous decline in *A. syriaca* N_e_ in the same study area during the past 600 years, which also increased in rate with the timing of widespread land clearing and urban development (Breitbart et al. in prep). The picture that emerges is that a combination of range expansions post-glaciation, combined with anthropogenic disturbance due to farming and urbanization are associated with rapid declines in N_e_ of each of the insect species examined.

### Effects of urbanization on population structure and gene flow

We did not find any clear effect of urbanization on differentiation among populations or population genetic structure. These results imply that urbanization does not influence gene flow of the insect species studied. We expected that monarchs would have the lowest differentiation between sampled populations as it has the highest dispersal distance. While we did see lower F_ST_ values in monarch populations compared to the weevil and beetle populations, and these values are similar to previous continent-wide sampling of monarchs (e.g., [89,90]), there was no difference between urban and rural populations. While this gives some support that dispersal distance influences population structure, we did not find a significant difference between the weevil and beetle populations. This may be due to a possible threshold of dispersal distance which mitigates population structure that is above both the weevil and beetle dispersal distance. However, monarchs have high dispersal ability and produce offspring along their migration route [91], whereas the weevil and beetle are residents in their northernmost range, which may explain the disparity between these species.

Furthermore, we expected that at least the weevil, the least dispersive species, would show greater differentiation in cities. Weevil populations had greater F_ST_ values than the other species, and F_ST_ among urban populations was two times greater than F_ST_ among rural populations in the largest city of Toronto, but there was large variation, and these trends were not significant. We see three possible explanations for the lack of any pattern. First, Toronto is a relatively young city, and it is possible that there has simply not been enough time for differentiation to occur. Second, differentiation is typically dependent on spatial scale (i.e., isolation-by-distance), and our sampling was simply not sufficiently large to test whether the spatially dependent differentiation among species differed among habitats. Third, the depletion of genetic variation noted in the previous section may limit the amount of genetic variation in populations, making it difficult for differentiation to evolve. We cannot distinguish these possible explanations, but it is clear that the specialist herbivores of milkweed do not support either the urban fragmentation or urban facilitation hypotheses proposed earlier.

As with genetic diversity, our results contrast with previous literature. A previous quantitative review found that urbanization influenced population differentiation in 85% of cases examined, with 66% of studies finding greater differentiation among urban populations. A classic example of this pattern are red-backed salamanders in Montreal, which showed greater differentiation among populations in older parts of the city, and little differentiation outside of the city [92]. However, 19% of studies found the opposite result, such as black-widow spiders where there is greater connectedness among populations within cities than in natural habitats, and there is even evidence of long-distance dispersal between cities [93]. Our study provides a counterexample to these previous studies.

## Conclusions

Our study shows that long-term natural and anthropogenic patterns likely explain the demographic history of populations, but that urbanization shows no clear effects on genetic drift and gene flow, and the ensuing patterns of genetic diversity and differentiation in three milkweed specialist insects. Our results suggest that post-glacial dispersal of the host plant and focal specialist herbivores to range margins in Ontario, followed by severe large scale anthropogenic disturbance, has depleted genetic variation within populations to such a degree that there is little genetic variation on which urbanization can influence non-adaptive processes. This study illustrates the importance of how the historical context of species can influence their ability (or lack thereof) to respond to historical and contemporary anthropogenic disturbance such as urbanization.

## Supporting information

S1 Fig Stairway plots with confidence intervals. Stairway plots from top to bottom for monarch, beetle, and weevil. Solid lines are for rural samples and dotted lines are for urban samples, shaded areas are 95% confidence intervals.
(TIF)

S2 Fig Rate of change of Ne over time for the monarch, weevil and beetle. Top panel: Rate change over the past 50000 years. Bottom panel: Rate change for just the past 2000, showing the accelerating loss of Ne per year, especially during the past 200–400 years. Rate change was calculated as the first-derivate of Ne change over time (per year).
(TIF)

S3 Fig PCAs colored by city for each species. To identify potential clustering by city that is not easily viewed in the PCA, each panel is separated from left to right by the city sampled and from top to bottom by the species. The outliers have been removed for monarchs. There is no clear cluster separation of habitat type within cities or across cities.
(TIF)

S4 Fig Barplots showing ADMIXTURE results at K = 2–6 for each of the three species. The most supported K value is highlighted in gray, with monarch and weevil at K = 2 and beetle K = 5. For each city, samples are in order from rural to urban, with a black bar between the two habitat types. There were no urban samples for the beetle in Kitchener and no urban samples for the weevil in Kitchener or London. With the best supported K values, there is no clear city or habitat type separation of clusters.
(TIF)

S1 Table Characteristics of each of the sampled cities. For each city, we indicate the latitude and longitude of the population closest to the city center, the number of sites (N), the human population size, city area (km^2^), population density (people/km^2^), and the total number of dwellings. All data describing city characteristics were taken from Statistics Canada’s 2016 Census data (http://www12.statcan.gc.ca/census-recensement/2016/dp-pd/index-eng.cfm).
(TIF)
