# Supplementary figures and images for: No detectable effect of urbanization on genetic drift or gene flow in specialist herbivorous insects of milkweed
Source: PLoS One. 2025 Feb 14;20(2):e0318956. doi: 10.1371/journal.pone.0318956 (PMC11828359; doi:10.1371/journal.pone.0318956)

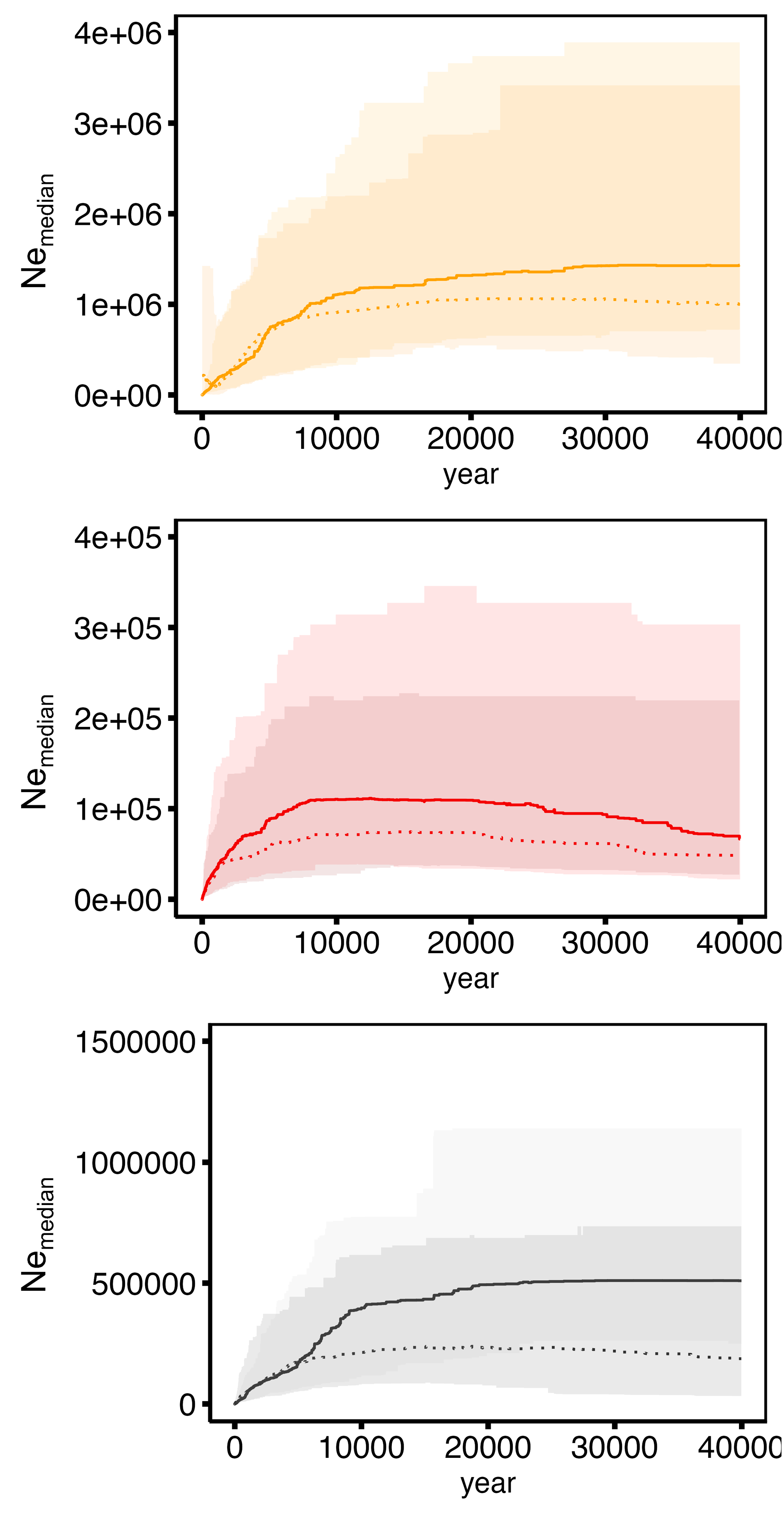

Supplement: S1 Fig — Stairway plots from top to bottom for monarch, beetle, and weevil. Solid lines are for rural samples and dotted lines are for urban samples, shaded areas are 95% confidence intervals. (TIF) [file pone.0318956.s001.tif]

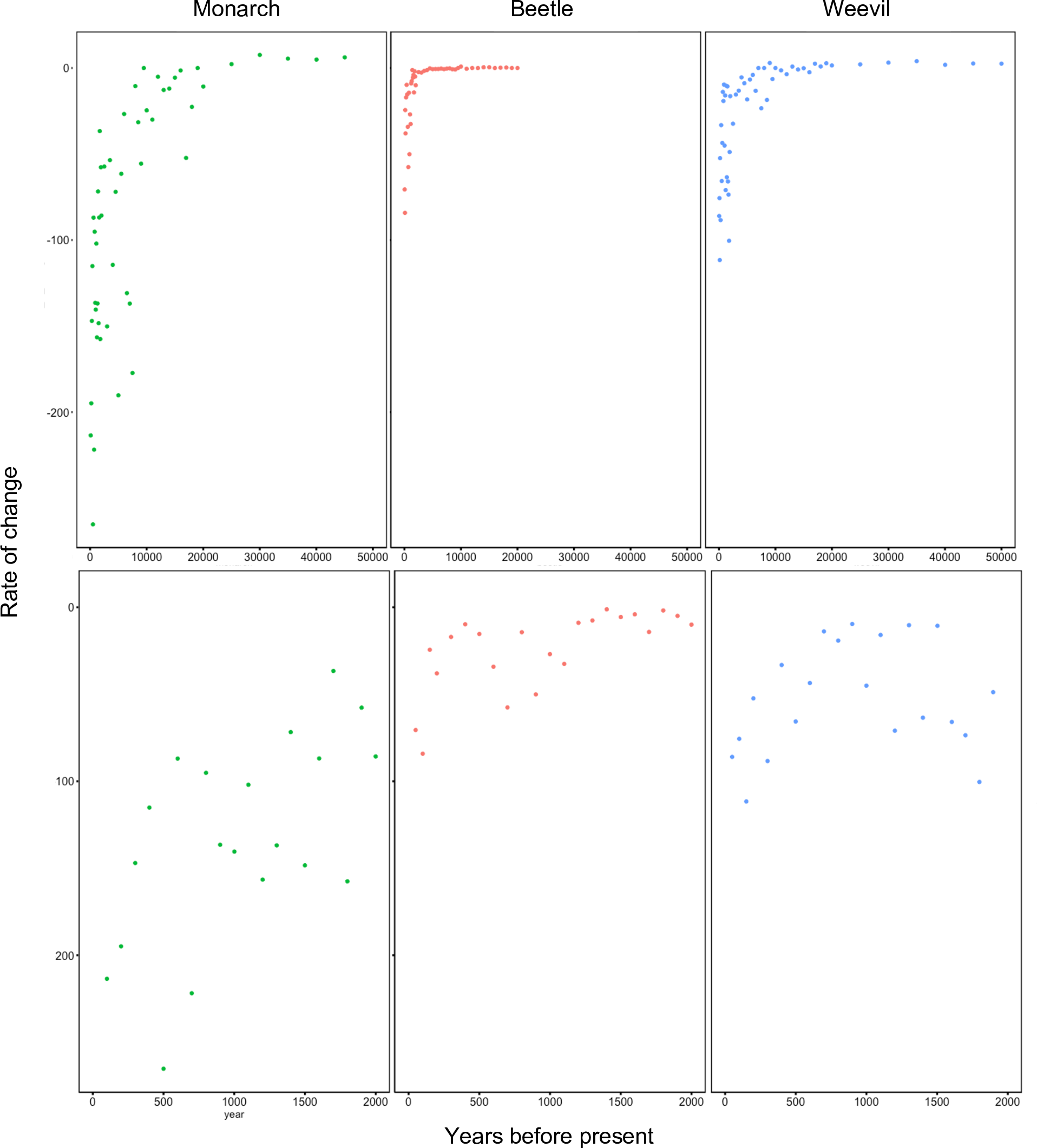

Supplement: S2 Fig — Top panel: Rate change over the past 50000 years. Bottom panel: Rate change for just the past 2000, showing the accelerating loss of Ne per year, especially during the past 200–400 years. Rate change was calculated as the first-derivate of Ne change over time (per year). (TIF) [file pone.0318956.s002.tif]

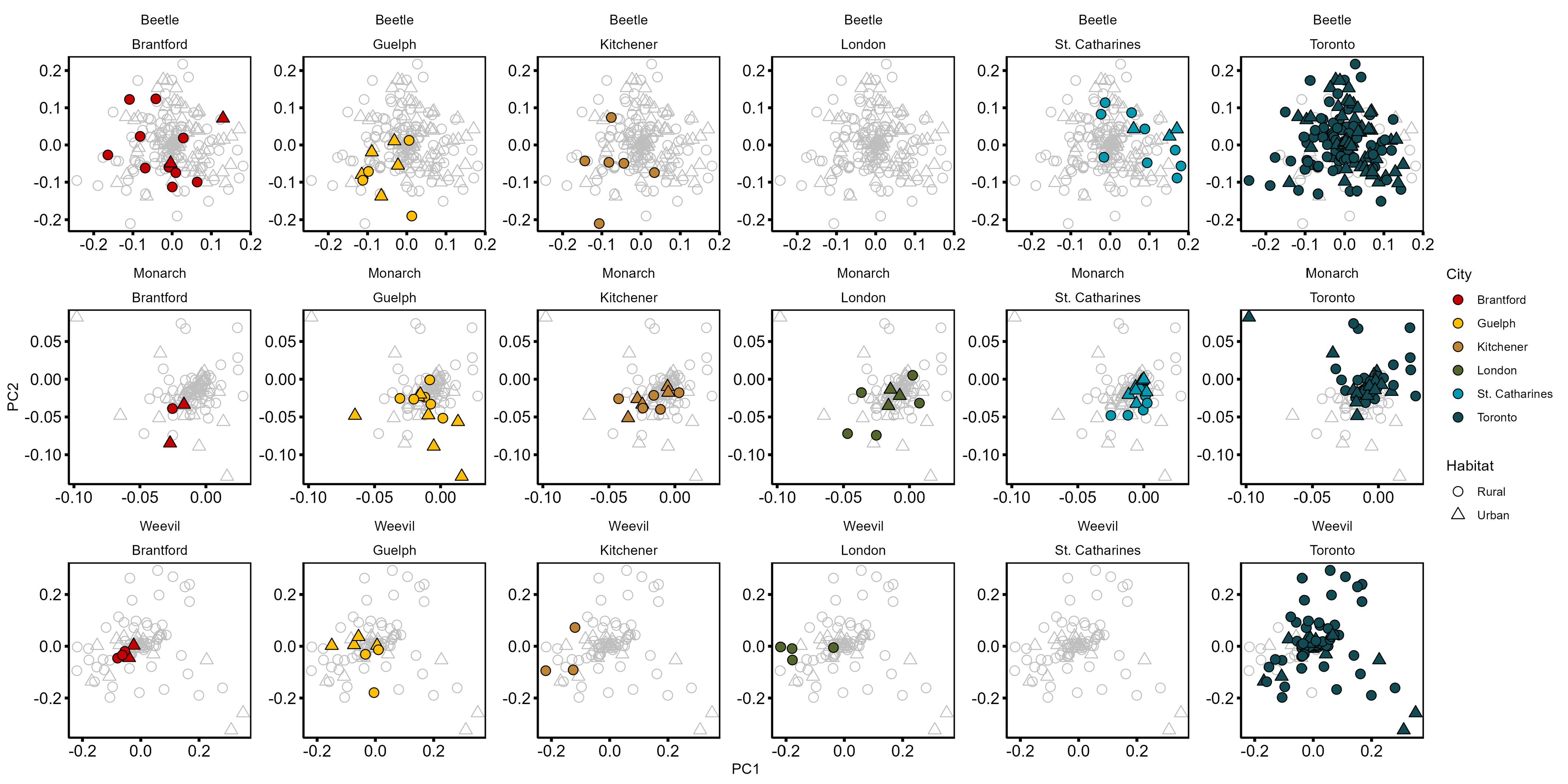

Supplement: S3 Fig — To identify potential clustering by city that is not easily viewed in the PCA, each panel is separated from left to right by the city sampled and from top to bottom by the species. The outliers have been removed for monarchs. There is no clear cluster separation of habitat type within cities or across cities. (TIF) [file pone.0318956.s003.tif]

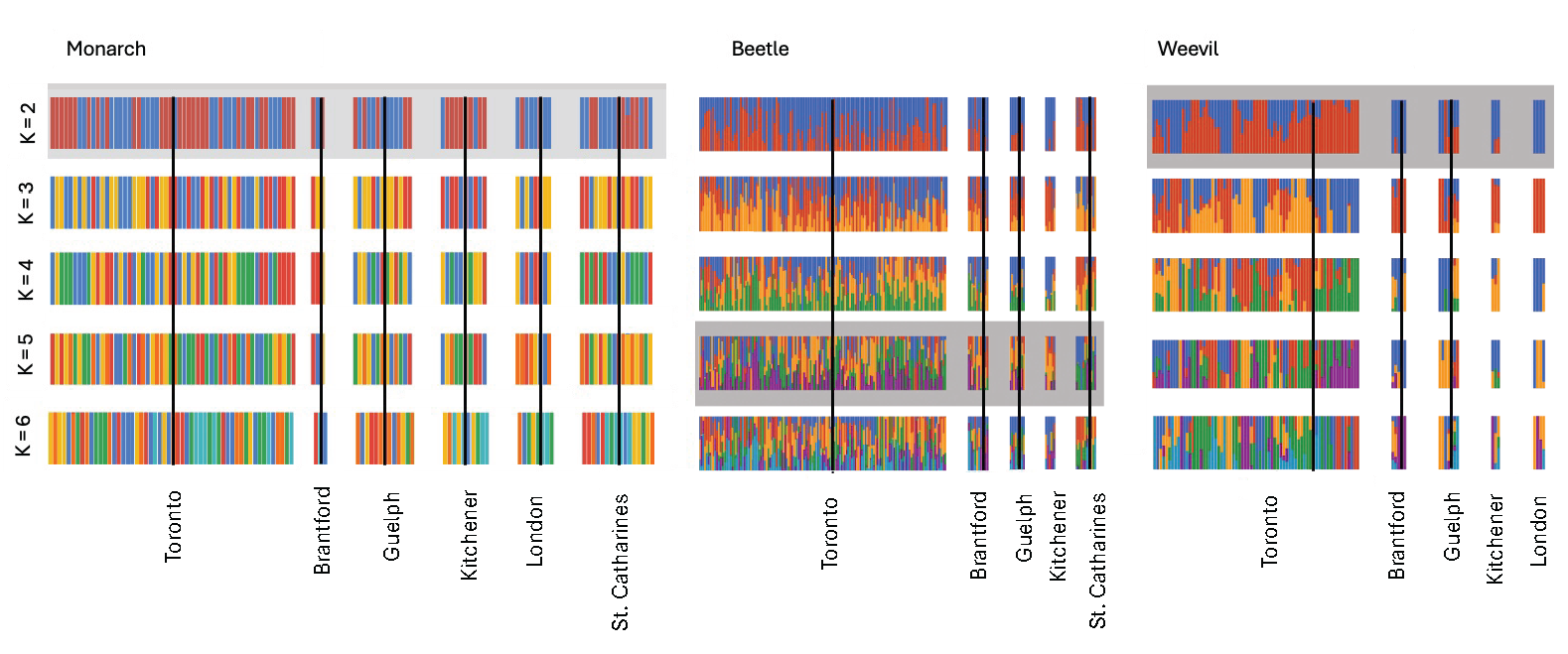

Supplement: S4 Fig — The most supported K value is highlighted in gray, with monarch and weevil at K = 2 and beetle K = 5. For each city, samples are in order from rural to urban, with a black bar between the two habitat types. There were no urban samples for the beetle in Kitchener and no urban samples for the weevil in Kitchener or London. With the best supported K values, there is no clear city or habitat type separation of clusters. (TIF) [file pone.0318956.s004.tif]

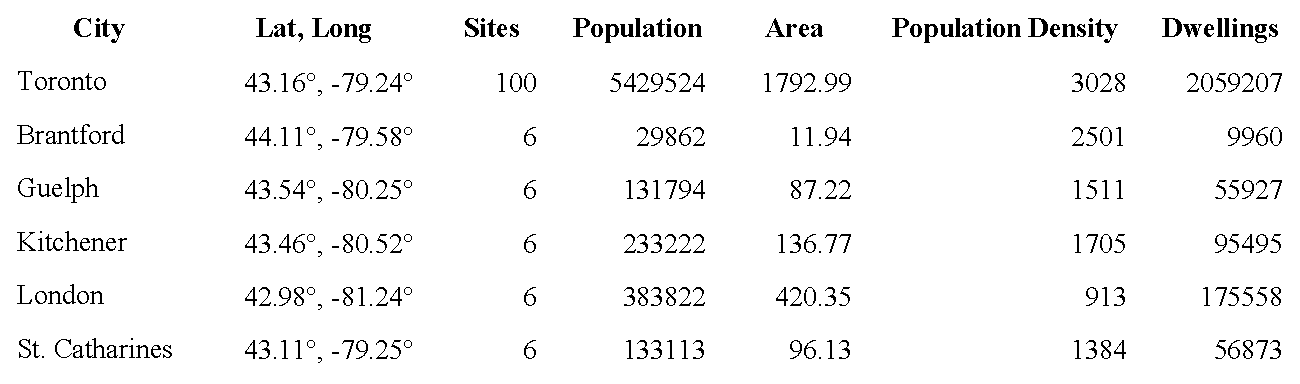

Supplement: S1 Table — For each city, we indicate the latitude and longitude of the population closest to the city center, the number of sites (N), the human population size, city area (km2), population density (people/km2), and the total number of dwellings. All data describing city characteristics were taken from Statistics Canada’s 2016 Census data (http://www12.statcan.gc.ca/census-recensement/2016/dp-pd/index-eng.cfm). (TIF) [file pone.0318956.s005.tif]
